# Supplementary material for: Application of an interpretable machine learning model based on optimal feature selection for predicting triple-vessel coronary disease: a multicenter retrospective study
Source: PeerJ. 2025 Dec 9;13:e20423. doi: 10.7717/peerj.20423 (PMC12700116; doi:10.7717/peerj.20423)
Supplement: Supplemental Information 3 [file peerj-13-20423-s003.docx]

| **中文原文** | **English Translation** |
| --- | --- |
| # 多核并行 | # Multi-core parallel processing |
| # 读取数据 | # Read data |
| # 修正变量类型 | # Correct variable types |
| # 将分类变量转换为factor | # Convert categorical variables to factors |
| # 删除无关变量在此处进行 | # Remove irrelevant variables here |
| # 删除含有缺失值的样本在此处进行，填充缺失值在后面 | # Remove samples with missing values here; imputation will be done later |
| # 数据概况 | # Data overview |
| # 设定阳性类别和阴性类别 | # Define positive and negative classes |
| # 转换因变量的因子水平，将阳性类别设定为第二个水平 | # Convert dependent variable levels and set the positive class as the second level |
| # 数据拆分 | # Split dataset |
| # 重抽样设定-5折交叉验证 | # Resampling setting - 5-fold cross-validation |
| # 数据预处理配方 | # Data preprocessing recipe |
| # 设定模型 | # Define model |
| # 贝叶斯优化超参数 | # Bayesian optimization for hyperparameters |
| # 交叉验证结果 | # Cross-validation results |
| # 图示 | # Visualization |
| # 经过交叉验证得到的最优超参数 | # Optimal hyperparameters obtained through cross-validation |
| # 采用最优超参数组合训练最终模型 | # Train the final model using the optimal hyperparameters |
| # 训练集预测评估 | # Model evaluation on training set |
| # 预测评估测试集预测评估 | # Model evaluation on test set |
| # ROC比较检验 | # ROC comparison test |
| # 合并训练集和测试集上ROC曲线 | # Combine ROC curves for training and test sets |
| # 合并训练集和测试集上PR曲线 | # Combine PR curves for training and test sets |
| # 合并训练集和测试集上校准曲线 | # Combine calibration curves for training and test sets |
| # 合并训练集和测试集上性能指标 | # Combine performance metrics for training and test sets |
| # 最优超参数交叉验证的结果 | # Cross-validation results using the optimal hyperparameters |
| # 保存评估结果 | # Save evaluation results |
